# Procedural outcome of lead explant and countertraction‐assisted femoral lead extraction in Thai patients with cardiac implantable electronic device infection

**DOI:** 10.1002/joa3.12574

**Published:** 2021-08-05

**Authors:** Jirarat Jiratham‐Opas, Narawudt Prasertwitayakij, Teerapat Nantsupawat, Wanwarang Wongcharoen

**Affiliations:** ^1^ Department of Internal Medicine Faculty of Medicine Chiang Mai University Chiang Mai Thailand

**Keywords:** CIED infection, countertraction‐assisted femoral lead extraction, lead removal

## Abstract

**Background:**

Cardiac implantable electronic device (CIED) implantation rate has been increasing worldwide. Despite proper surgical technique and preincisional intravenous antibiotics, the incidence of infected CIED remains high and leads to serious complications. When encountered with CIED infection, complete CIED system removal is indicated. Several lead extraction approaches have shown a high success rate. However, the facilities are limited in Thailand. In our current practice, we perform lead extraction using the Dotter basket snare femoral approach as our primary method. There are no prior data on this countertraction‐assisted transfemoral technique. Therefore, we aim to study the procedural outcome of countertraction‐assisted transfemoral lead removal technique of CIED infection in Thai patients.

**Methods:**

Patients diagnosed with CIED infection and with a history of device infection were retrospectively included. Simple manual removal was performed. In case of failure, we proceeded with the modified countertraction‐assisted transfemoral technique.

**Results:**

There were 35 patients in the study. The success rate was 94.3%. Most of the leads, 62.8%, were removed by simple manual traction. In the 37.1% who required further femoral approach lead extractions, procedural failure was observed in 5.7% and procedure‐related adverse events in 5.6%. CIED infection‐related death accounted for 5.7% and nosocomial infection‐related death, 2.8%.

**Conclusion:**

The success rate of CIED infection lead explant and countertraction‐assisted transfemoral lead extraction technique was high with small complications and can be performed without advanced facilities. However, the procedure required a main center with a cardiovascular thoracic surgery support team.

## Graphical abstract

Countertraction is the cornerstone of the lead extraction in preventing of right ventricular (RV) inversion with less complications. Improvised technique, using telescopic system consisted of ablation catheter and deflectable sheath placement at RV apex, provided countertraction force whilst applying femoral lead extraction.

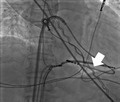

## 1 INTRODUCTION

Cardiac implantable electronic device (CIED) implantation rate has been continuously increasing worldwide.^1^, ^2^ Despite proper skin antiseptic, surgical technique, and preincisional intravenous antibiotics, the incidence of infected CIED remains high at 1%‐2%.^3^, ^4^ CIED infection is one of the most serious cardiac device complications because of the worsening quality of life, life‐threatening condition, prolonged hospitalization, risk of device removal, and high mortality rate.^5^, ^6^, ^7^, ^8^ On CIED infection, complete CIED system removal is indicated.^9^ Transvenous lead extraction can be performed by the superior, inferior (femoral), or combined approach. Numerous tools and techniques are available, for example, simple manual traction, locking stylets, telescoping sheaths, snares, mechanical cutters, and laser sheaths.^10^ However, the locking stylets, telescoping sheaths, powered tools, and Needle's Eye Snare have limited availability in many regions of Thailand because of reimbursement and cost issues, including our center. Therefore, we have been mainly using the Dotter basket snare for that has adequate tensile strength for lead extraction. Through a long deflectable sheath, we placed an ablation catheter in the right ventricle to provide countertraction and prevent right ventricle (RV) inversion. Given the paucity of data on this modified RV support countertraction‐assisted transfemoral lead extraction, we studied the safety and efficacy of this approach in patients with CIED infection.

## 2 METHODS

In this retrospective descriptive single‐center study, we included all patients diagnosed with CIED infection who had undergone device removal after obtaining informed consent. The patients who underwent CIED removal at the university hospital (Maharaj Nakorn Chiang Mai Hospital, Thailand) between January 2007 and March 2020 were included. Patient information was collected from the electronic medical record system (Digicard software^®^). Baseline characteristics including age, gender, comorbidities, indication of CIED implantation, targeted leads including their type, the type of device, targeted lead dwell time, and procedure time were collected.

### 2.1 Definition

#### 2.1.1 Definition of cardiac implantable electronic device infection

In accordance with previous studies,^10^, ^11^ CIED infection was categorized as follows: (a) isolated generator pocket infection, (b) isolated pocket erosion, (c) pocket site infection, (d) lead infection, (e) systemic inflammatory response with or without pulse generator pocket involvement, or (f) fever of undefined origin with positive blood cultures, especially staphylococcal species.

#### 2.1.2 Definition of early, late, or delayed CIED infection

Early, late, or delayed CIED infection is defined as infection that occurred within 30 days, between 30 and 364 days, and more than 364 days after device implantation, respectively.^10^, ^12^, ^13^

#### 2.1.3 Lead dwell time

Lead dwell time is the time from device implantation to time of device removal.

#### 2.1.4 Procedural details

**FIGURE 1:**
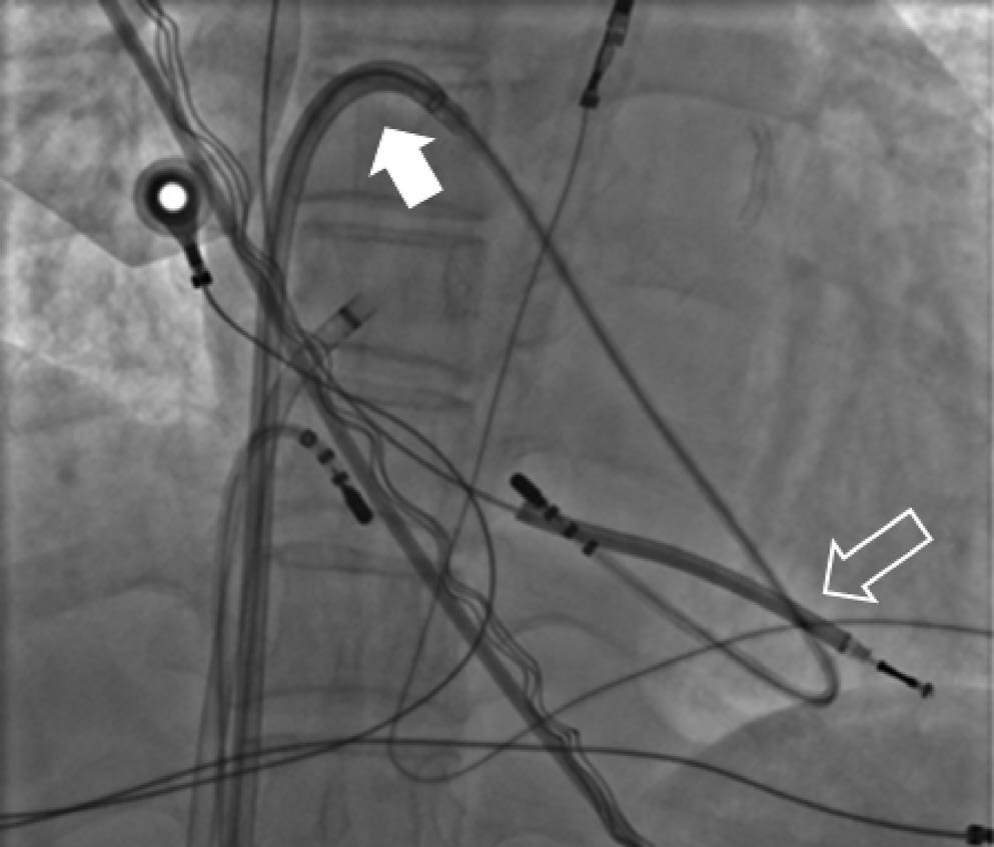
RAO view showing a 4‐mm tip ablation catheter through steerable sheath looped into the right ventricular (RV) apex forming primary (open arrow) and secondary curves (close arrow) of the telescopic countertraction system

**FIGURE 2:**
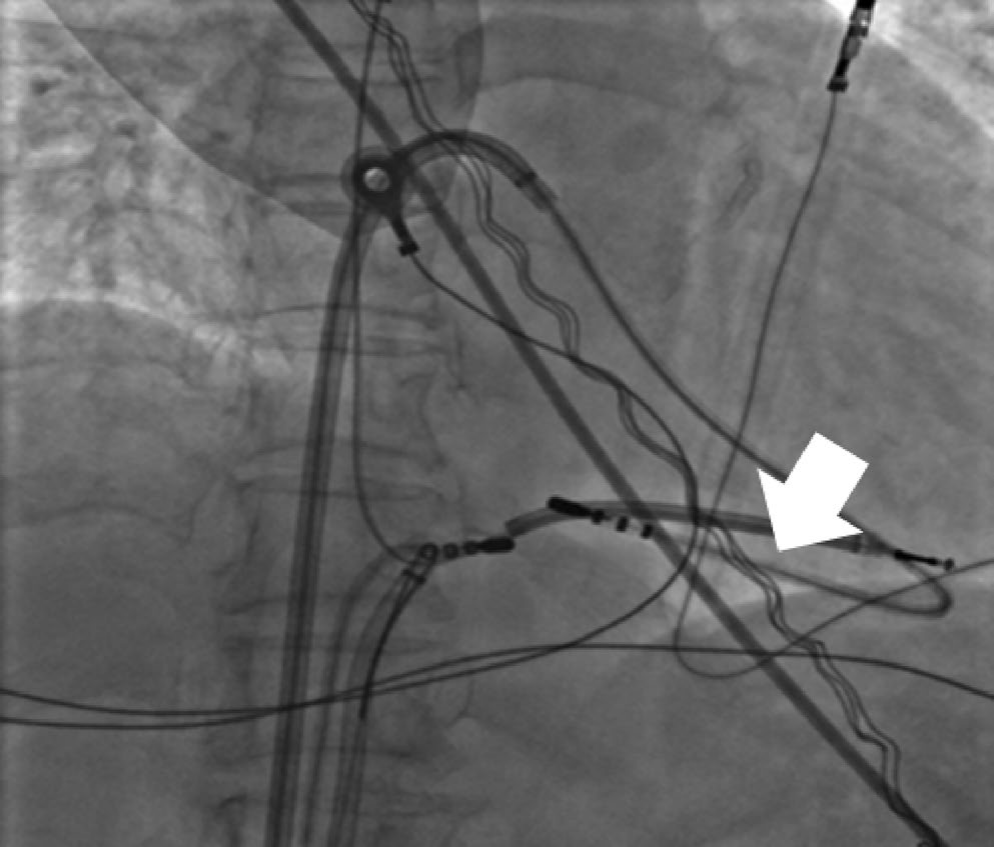
RAO view showing a second 4‐mm tip ablation catheter, from short vascular sheath, advanced over the target defibrillator lead (white arrow). The Dotter basket snare, via the remaining steerable sheath, was then used to grasp the ablation catheter shaft from another side, which ensured capturing the target lead

**FIGURE 3:**
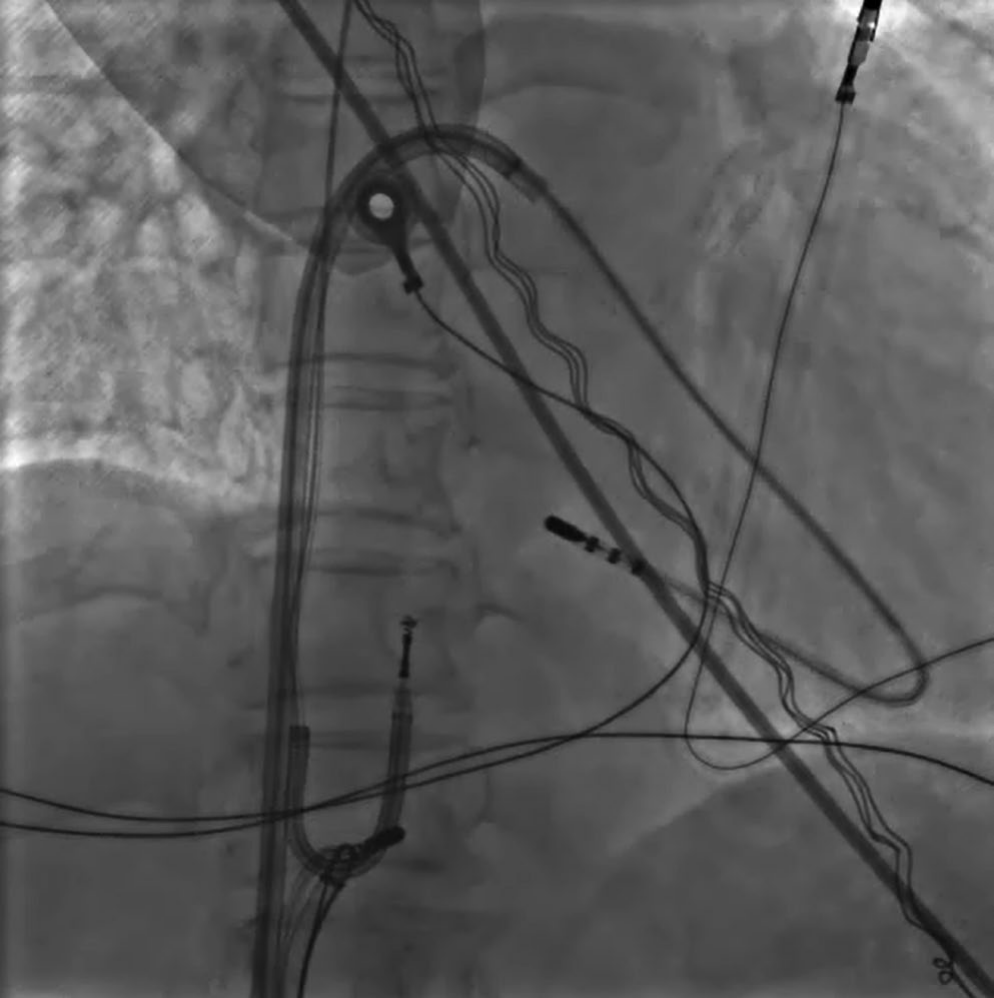
RAO view showing that the target lead was pulled down through which the distal lead tip was freed from the myocardium. Countertraction system stayed at their positions

The device was removed under local anesthesia and controlled sedation. Following opening of the device pocket and discharge of the debris, we performed simple manual traction (explant) in all patients through the superior approach at the lead insertion site. If the targeted lead(s) could not be freely removed by simple manual traction with or without using a standard stylet (also mentioned as “explant”), we proceeded to a femoral approach (extraction) by placing one 8‐French short sheath and two steerable long sheaths (8.8 F Agilis NxT Steerable Introducer 71 cm, Abbott Cardiovascular System Inc.; formerly St. Jude Medical) into the right femoral vein. We inserted a nonirrigated 4 mm tip ablation catheter through the first steerable sheath, and looped it into the right ventricular (RV) apex to prevent RV inversion, as primary curve, and bent steerable sheath creating a secondary curve (Figure 1). A second nonirrigated 4 mm tip ablation catheter was inserted through a 8‐French short sheath and the Dotter basket snare through the remaining steerable sheath. We used this second ablation catheter as a threader passing from one side of the target lead, which is then grasped with the Dotter basket snare that is passed from the other side (Figure 2). The lead is then pulled down until the distal lead tip is freed from the RV myocardium (Figure 3). The freed lead is then recaptured with the same snare without threader and pulled further into the countertraction sheath, before lead cutting is made, which allowed complete lead removal be made with transfemoral through deflectable sheath and via the pectoral pocket. Movie files are include in this article as Video S1 and S2. A similar procedure was used for the right femoral vein.

#### 2.1.5 Definition of complete procedural success

Complete procedural success is defined as removal of all targeted leads and material, with the absence of any permanently disabling complication or procedure‐related death.^10^

#### 2.1.6 Definition of procedural failure

Procedural failure is defined as lead extraction procedures in which complete procedural or clinical success cannot be achieved, or the development of any permanently disabling complication, or procedure‐related death.^10^

Clinical success is defined as lead extraction procedures with removal of all targeted leads and lead material from the vascular space or retention of a small portion of the lead (4 mm) that does not negatively impact the outcome goals of the procedure.

#### 2.1.7 Definition of adverse events

Adverse events involve procedural complications and nonprocedural complications described previously.^10^, ^11^

*Procedural complications* are events that span the time the patient enters the operating room and following the procedure.

*Major complications* are those that pose an immediate threat to life or that result in death, unexpected events that cause persistent or significant disability or any event that requires significant surgical intervention.

*Minor complications* are undesired adverse events that require medical intervention, including minor procedural interventions, but do not significantly affect the patient's function, nor does it threaten life or cause death.

### 2.2 Statistical analysis

**FIGURE 4:**
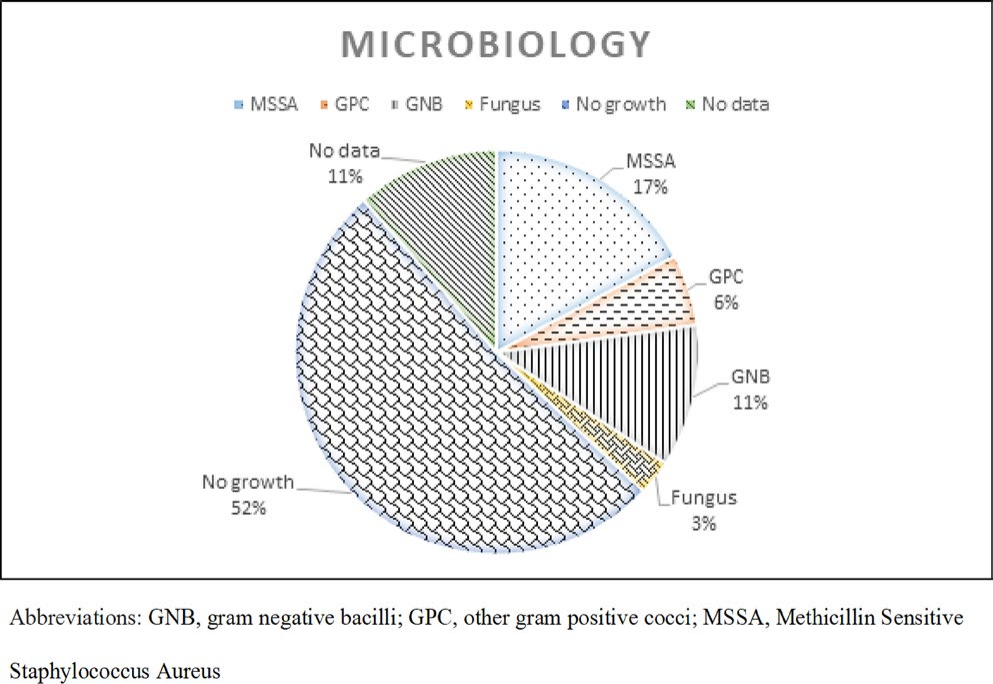
Microbiological data

Continuous variables are presented as mean ± SD or median ± interquartile range when appropriate. Categorical variables are displayed as percentages. Frequency distribution is shown as numerical values and graph (Figure 4).

## 3 RESULTS

There were 35 patients who had CIED infection and device removal at our university hospital between January 2007 and March 2020. The mean age of the patients was 64.4 ± 13.5 years with male predominance (65%). The two most common CIED infections were dual‐chamber permanent pacemaker (14 cases, 40%) and automated implantable cardioverter defibrillator (AICD) (10 cases, 28.5%). Most of the CIED infections occurred >364 days post implant (delayed type) in 19 cases (54.3%) (Table 1). The mean procedure time was 35 ± 15 and 104 ± 65 minutes in the explant and femoral extraction groups, respectively. Of the 56 leads, 39 leads (70%) required simple manual traction or explant and 17 leads (30%) required further transfemoral extraction technique. The mean dwell time of targeted leads was 24 ± 29 and 105 ± 48 months in the explant and femoral extraction groups, respectively (Table 2).

**TABLE 1:** Baseline characteristics

Baseline characteristics	Total (N = 35)
Age, years	64.4 ± 13.5
Gender (M:F)	23:12 (64:35%)
Comorbidities	
Coronary artery disease	6 (17%)
Heart failure with reduced ejection fraction	11 (30%)
Atrial fibrillation	12 (33%)
Device indication	
Sick sinus syndrome	14 (41%)
Atrioventricular block	19 (55%)
Primary SCD prevention	2 (5.8%)
Secondary SCD prevention	7 (20.5%)
Types of CIED infection	
Single‐chamber pacemaker	5 [14.3%]
Dual‐chamber pacemaker	15 [42.9%]
AICD	10 [28.6%]
CRT	2 [5.7%]
Pulse generator change	2 [5.7%]
Upgrade procedure	1 [2.8%]
CIED infection types	
Early (<30 days postimplant)	8 [22.8%]
Late (30‐364 days postimplant)	8 [22.8%]
Delay infection (>364 days postimplant)	19 [54.3%]
Target leads	56 leads
Atrial pacing leads	19 leads
RV pacing leads	22 leads
AICD dual coil	10 leads
AICD single coil	3
CS leads	2 leads

Note

Values are mean ± SD or n (%).

Abbreviations: AICD, automatic implantable cardioverter defibrillator; CRT, cardiac resynchronization therapy; CS, coronary sinus; RV, right ventricle; SCD, sudden cardiac death.

**TABLE 2:** (A) Procedural and (B) clinical lead success and failure rate

(A) Procedural success and failure rate	
Procedural outcomes	Total (N = 35)
Complete procedural success	33 (94.3%)
Procedural failure	2 (5.7%)
Procedure time	Minutes [mean ± SD]
Explant	13‐60 [35 ± 15]
Extraction	42‐240 [104 ± 65]
Complete lead removal	Total leads 56 leads
Atrial lead (19 leads)	19 leads (100%)
RV pacing lead (22 leads)	22 leads (100%)
AICD dual coil (10 leads)	8 leads (80%)
AICD single coil (3 leads)	3 leads (100%)
CS lead (2 leads)	2 leads (100%)
Lead dwell time	Month (mean ± SD)
Explant	1‐70 [20 ± 24]
Extract	52‐169 [88 ± 35]

(B) Clinical characteristic by lead success and lead failure				
Lead type (N = 56)	Explant (N = 39)		Extraction (17)	
	Success	Failure	Success	Failure
RA pacing lead (N = 19 leads)	15 (27%)	—	4 (7%)	—
RV pacing lead (22 leads)	13 (23%)	—	9 (16%)	—
AICD dual coil (8 leads)	6 (11%)	—	2 (4%)	—
AICD single coil (5 leads)	3 (5%)	—	—	2 (4%)
CS 2 leads	2 (4%)	—	—	—

Abbreviations: AICD, automatic implantable cardioverter defibrillator; CRT, cardiac resynchronization therapy; CS, coronary sinus; RV, right ventricle.

The overall procedural success rate was 94.3% (33 of 35). All of the procedures were performed under local anesthesia. Most of the procedures required simple manual traction technique (22 patients, 62.8%). There were 13 patients (37.1%) who required further femoral approach lead extractions. The overall procedural failure occurred in two patients (5.7%), which recapitulated as 96% (54 of 56 leads) and 4% (2 of 56 leads) of total successful and failure lead removal rate (Table 2). The first procedural failure occurred in a 43‐year‐old male with Brugada syndrome with AICD lead removal failure, because of the tear of goose neck snare, which required surgical thoracotomy for AICD lead removal. Initially, a goose neck snare was used, but later it was substituted with a Dotter basket snare to match with the required tensile strength. Another procedural failure occurred in a 68‐year‐old male with ischemic cardiomyopathy with AICD implantation. There was RV inversion, with compromised hemodynamics during AICD lead extraction, caused by fluoroscopic‐confirmed suboptimal position of RV countertraction catheter. The operator immediately altered the RV catheter and its steerable sheath into the apex, which successfully reversed the inverted RV myocardium. The patient was transferred to a cardiac intensive care unit for hemodynamic stabilization and close monitoring. The target ICD lead was abandoned in the inferior vena cava (IVC), since the cardiac surgeon considered that surgical lead removal via open thoracotomy was not justified. For the difficult RA and CS lead removal, all leads were detached from the fibrotic tissue after applying the method mentioned previously. There were four RA leads that required extraction. There was no complication in removing the RA and CS leads including atrial avulsion, tricuspid valve, or CS injury.

There were two (5.7%) procedure‐related adverse events including one patient with right femoral vein injury, after successful removal, requiring local surgical repair and one patient with RV inversion (Table 3). There was no any atrial avulsion, tricuspid valve or coronary sinus injury during RA or CS leads removal.

**TABLE 3:** Clinical characteristics of failure cases

	Case 1	Case 2
Age, years	43	68
Gender	Male	Male
Types of device	AICD for secondary prevention	AICD for secondary prevention
Underlying diseases	Brugada syndrome	Ischemic cardiomyopathy, paroxysmal atrial fibrillation, essential hypertension, CKD stage IV
Implant time, months	43	65

Abbreviations: AICD, automatic implantable cardioverter defibrillator; CKD, chronic kidney disease.

### 3.1 Nonprocedure‐related complications

There were two (5.7%) CIED infection‐related deaths. One patient died from *Staphylococcus aureus* sepsis with multiorgan failure. Another patient had CIED infection complicated with infected CAPD and candida sepsis. There was one (2.8%) nosocomial infection‐related death (from pneumonia). Two cases (5.7%) developed CIED infection‐related systemic infection (Table 3). The most prevalent organisms were methicillin‐sensitive *S. aureus* (6 cases, 16.7%) (Table 4).

**TABLE 4:** Adverse events

Adverse events	n (%)
Procedure‐related complications	
Right femoral vein laceration	1 (2.8)
Right ventricle inversion	1 (2.8)
Non‐procedure‐related complications	
CIED infection‐related death	2 (5.7)
Nosocomial infection‐related death	1 (2.8)
CIED‐related systemic infection	2 (5.7)

Abbreviation: CIED, cardiovascular implantable electronic device.

## 4 DISCUSSION

Complete device and lead removal is crucial for all patients with definite CIED infection. Lead extraction is the most serious procedure of lead removal. In addition to lead explant, lead extraction by femoral approach is one of the major procedural approaches. In most of the procedures, the leads could be removed by explant including most of atrial and CS leads. About one‐third of our cases required further transfemoral extraction technique. Not all of the leads in the transfemoral extraction group required transfemoral extraction technique; however, the cases which required transfemoral extraction technique showed longer lead dwell time, procedural time, and were associated with more complications. Starck et al^14^ used a superior subclavian approach (SCA) and femoral access for accessing the lead scheduled for extraction. In case of failure of the SCA, a bailed out femoral snare approach was performed using the Needle’s Eye Snare device; this increased clinical success by 12.6% (from 83.7% to 96.3%). de Bie et al^15^ also performed lead extraction by manual traction and femoral approach using a variety of extraction tools including locking stylet and femoral snare without lead extraction sheaths. The clinical success increased from 84.8% to 93.5% using femoral snaring. Klug et al^16^ showed a success rate of 87.2%, operating Needle's Eye Snare via femoral approach concomitantly with countertraction on the distal fixation of the lead with a long 16 Fr sheath introduced via a femoral vein. Bracke et al^17^ reported utilizing needle eyes via femoral introducer sheath and 12 Fr sheath with a success rate of 94.4%. Compared with previous studies, our study had no advanced equipment or facilities. In this study, the success rate increased from 62.8% to 94.2%. Our lead removal failure rate was 5.7% involving two patients. Either the countertraction ablation catheter was sub optimally placed at RV apex or using goose neck snare, which provided inadequate countertraction force or inadequate tensile strength were identified as major causes of failure in the two cases. The two procedures were undertaken in 2013 and 2015, which were the beginning year of our RV support counetrtraction‐assisted transfemoral lead extraction. This failure has never been observed after technical refinement with decent basket snare system and proper RV support location, which eventually yield 100% successful in atrial, RV and LV lead removal and 88% in AICD lead removal.

In the Stack et al^14^ study, major complications were encountered in two patients (1.7%) with RV avulsion and asystole during lead extraction procedure. Minor complications (3.5%) included pocket hematomas requiring surgical drainage and pneumothorax requiring chest tube placement. Using transvenous lead removal without the use of extraction sheaths, de Bie et al^15^ found 0.7% major complications including atrial rupture and cardiac tamponade, and tricuspid regurgitation that were treated surgically and 4.7% minor complications including pocket hematoma, pneumothorax requiring drainage, and lead migration to the pulmonary vasculature. Klug et al^16^ showed two deaths and one transient ischemia of the right inferior limb. Our study showed small numbers, two cases (5.5%) of procedure‐related adverse events. There were one major (2.8%) and one minor procedure‐related adverse event (2.8%). The major adverse event was one RV inversion as the countertraction RF ablator was in a suboptimal position at the RV apex with unstable hemodynamic. The procedure was terminated earlier with abandoned lead in the IVC; however, overall clinical conditions were stable after the RV reversion. Also, another adverse event was a minor complication with right femoral vein injury after complete procedure requiring surgical repair; however, local femoral vein repair was performed at the end of the procedure and no extensive surgery was required. We had no procedure‐related death, procedure‐related cardiac valve damage, venous lacerations, lead tip fracture that required surgical intervention, cardiac perforation or cardiac tamponade, venous thromboembolism event, or pneumothorax.

### 4.1 Limitations

The limitations of this study are retrospective design and single‐center experience. Further limitations include the uncontrolled nature of the data, small sample size, and some missing data.

Although our complication rate was small (5.6%), rate of complications may be differ in different lead dwell time, lead position, propreity of RV countertraction, in which lead to higher rate of RV inversion, atrial avulsion, tricuspid valve or CS injury.

Because of the small sample size, our study showed a different mean lead dwell time between the explant and extraction groups. The mean lead dwell time in the extraction group was 88 months compared with 20 months in the explant group. Furthermore, our study showed that the shortest lead dwell time in the explant group was 52 months, whereas lead dwell time in the explant group ranged between 1 and 70 months. Unfortunately, there was no further analysis specifically within group with dwell time beyond 50 months.

Another issue is the re‐use of steerable and deflectable ablation catheters. We are also aware of recall and warning issues of resterilized Agilis^®^ sheath.

We used the Dotter basket snare because of its availability, robustness, and adequate tensile strength. Other snares with adequate tensile strength may be also used and taken into consideration.

We performed the extraction procedure with a standby cardiothoracic team, which may or may not be widely applicable. A final limitation is that we included only patients with CIED infection without available standard extraction procedure or not affordable, thereby strongly precluding its broad application.

## 5 CONCLUSION

RV countertraction‐assisted transfemoral lead extraction can be useful RV lead removal procedure in low volume extraction program without available lead locking stylet‐based procedures. Advantages include the user‐friendly nature, simple tools, and cost‐effectiveness. The operator has to be cognizant of the critical position of the countertraction assembly system technique and also of proper snaring tools for specific transfemoral lead extraction.

## CONFLICT OF INTEREST

Authors declare no conflict of interests for this article.

## Supporting information

Video S1

Video S2

## References

[1] Greenspon AJ, Patel JD, Lau E, Ochoa JA, Frisch DR, Ho RT, et al. 16‐year trends in the infection burden for pacemakers and implantable cardioverter‐defibrillators in the United States 1993 to 2008. J Am Coll Cardiol. 2011;58(10):1001–6. 21867833 10.1016/j.jacc.2011.04.033

[2] Lee JH, Lee S‐R, Choi E‐K, Jeong J, Park H‐D, You S‐J, et al. Temporal trends of cardiac implantable electronic device implantations: a nationwide population‐based study. Korean Circ J. 2019;49(9):841–52. 31074230 10.4070/kcj.2018.0444 PMC6713826

[3] Korkerdsup T, Ngarmukos T, Sungkanuparph S, Phuphuakrat A. Cardiac implantable electronic device infection in the cardiac referral center in Thailand: incidence, microbiology, risk factors, and outcomes. J Arrhythm. 2018;34(6):632–9. 30555607 10.1002/joa3.12123 PMC6288561

[4] Prutkin JM, Reynolds MR, Bao H, Curtis JP, Al‐Khatib SM, Aggarwal S, et al. Rates of and factors associated with infection in 200 909 Medicare implantable cardioverter‐defibrillator implants: results from the National Cardiovascular Data Registry. Circulation. 2014;130(13):1037–43. 25081281 10.1161/CIRCULATIONAHA.114.009081

[5] Baman TS, Gupta SK, Valle JA, Yamada E. Risk factors for mortality in patients with cardiac device‐related infection. Circ Arrhythm Electrophysiol. 2009;2(2):129–34. 19808457 10.1161/CIRCEP.108.816868

[6] Sohail MR, Henrikson CA, Braid‐Forbes MJ, Forbes KF, Lerner DJ. Mortality and cost associated with cardiovascular implantable electronic device infections. Arch Intern Med. 2011;171(20):1821–8. 21911623 10.1001/archinternmed.2011.441

[7] de Bie MK , van Rees JB , Thijssen J, Borleffs CJW, Trines SA, Cannegieter SC, et al. Cardiac device infections are associated with a significant mortality risk. Heart Rhythm. 2012;9(4):494–8. 22056722 10.1016/j.hrthm.2011.10.034

[8] Nery PB, Fernandes R, Nair GM, Sumner GL, Ribas CS, Divakara menon SM, et al. Device‐related infection among patients with pacemakers and implantable defibrillators: incidence, risk factors, and consequences. J Cardiovasc Electrophysiol. 2010;21(7):786–90. 20102431 10.1111/j.1540-8167.2009.01690.x

[9] Blomström‐Lundqvist C, Traykov V, Erba PA, Burri H, Nielsen JC, Bongiorni MG, et al. European Heart Rhythm Association (EHRA) international consensus document on how to prevent, diagnose, and treat cardiac implantable electronic device infections‐endorsed by the Heart Rhythm Society (HRS), the Asia Pacific Heart Rhythm Society (APHRS), the Latin American Heart Rhythm Society (LAHRS), International Society for Cardiovascular Infectious Diseases (ISCVID) and the European Society of Clinical Microbiology and Infectious Diseases (ESCMID) in collaboration with the European Association for Cardio‐Thoracic Surgery (EACTS). Europace. 2019;22(4):515–49. 10.1093/europace/euz246 PMC7132545 31702000

[10] Kusumoto FM, Schoenfeld MH, Wilkoff BL, Berul CI, Birgersdotter‐Green UM, Carrillo R, et al. 2017 HRS expert consensus statement on cardiovascular implantable electronic device lead management and extraction. Heart Rhythm. 2017;14(12):e503–e551. 28919379 10.1016/j.hrthm.2017.09.001

[11] Baddour LM, Epstein AE, Erickson CC, Knight BP, Levison ME, Lockhart PB, et al. Update on cardiovascular implantable electronic device infections and their management: a scientific statement from the American Heart Association. Circulation. 2010;121(3):458–77. 20048212 10.1161/CIRCULATIONAHA.109.192665

[12] Bongiorni MG, Kennergren C, Butter C, Deharo JC, Kutarski A, Rinaldi CA, et al. The European Lead Extraction ConTRolled (ELECTRa) study: a European Heart Rhythm Association (EHRA) registry of transvenous lead extraction outcomes. Eur Heart J. 2017;38(40):2995–3005. 28369414 10.1093/eurheartj/ehx080

[13] Perez AA, Woo FW, Tsang DC, Carrillo RG. Transvenous lead extractions: current approaches and future trends. Arrhythm Electrophysiol Rev. 2018;7(3):210–7. 30416735 10.15420/aer.2018.33.2 PMC6141917

[14] Starck CT, Caliskan E, Klein H, Steffel J, Falk V. Impact of a femoral snare approach as a bailout procedure on success rates in lead extractions. Interact Cardiovasc Thorac Surg. 2014;18(5):551–5. 24535091 10.1093/icvts/ivu005

[15] de Bie MK , Fouad DA, Borleffs CJ, van Rees JB , Thijssen J, Trines SA, et al. Trans‐venous lead removal without the use of extraction sheaths, results of >250 removal procedures. Europace. 2012;14(1):112–6. 21873627 10.1093/europace/eur269

[16] Klug D, Jarwe M, Messaoudene SA, Kouakam C, Marquie C, Gay A, et al. Pacemaker lead extraction with the Needle's Eye Snare for countertraction via a femoral approach. Pacing Clin Electrophysiol. 2002;25(7):1023–8. 12164441 10.1046/j.1460-9592.2002.01023.x

[17] Bracke FA, Dekker L, van Gelder BM . The Needle's Eye Snare as a primary tool for pacing lead extraction. Europace. 2013;15(7):1007–12. 23277531 10.1093/europace/eus426

